# Characterization of the Aromatic Profile of Mature-Green Tomato Fruit (‘Fen Junmei’) in Response to Supplemental LED Light Quality and Intensity During Ripening

**DOI:** 10.3390/ijms27219555

**Published:** 2026-10-26

**Authors:** Jingli Yang, Xiaohan Yu, Qingyu Wang, Ziyang Xia, Pan Yin, Xiaoyang Zhang, Jingmin Zhang, Byoung Ryong Jeong, Jinnan Song

**Affiliations:** 1 Weifang Key Laboratory for Stress Resistance and High Yield Regulation of Horticultural Crops, Shandong Provincial University Laboratory for Protected Horticulture, College of Jia Sixie Agriculture, Weifang University of Science and Technology, Shouguang 262700, China; yangjl940123@wfust.edu.cn (J.Y.); 17888362280@163.com (X.Y.); 15065628932@163.com (Q.W.); 19710016216@163.com (Z.X.); 19014550316@163.com (P.Y.); 19846876159@163.com (X.Z.); zhangjm303@wfust.edu.cn (J.Z.); 2 Department of Horticulture, Division of Applied Life Science (BK21 Four), Graduate School, Gyeongsang National University, Jinju 52828, Republic of Korea; brjeong@gnu.ac.kr; 3 Division of Horticultural Science, College of Agriculture and Life Sciences, Gyeongsang National University, Jinju 52828, Republic of Korea

**Keywords:** *Solanum lycopersicum*, light quality and intensity, chitosan oligosaccharide, postharvest ripening, terpenoid synthesis, tyrosine metabolism

## Abstract

Light quality and light intensity, as important environmental signals, can regulate the ripening process and flavor compound synthesis in tomato fruits. Chitosan oligosaccharides, as natural bio-stimulants, can improve postharvest quality of fruits and vegetables. However, how varied LED light quality–intensity regimes modulate ripening and aroma metabolism in chitosan-oligosaccharide-pre-treated mature-green tomato fruit remains unclear. This study used mature-green tomato fruits as experimental material, which were pre-treated by immersion in chitosan oligosaccharide solution. Subsequently, different combinations of supplemental light qualities (white light, blue light, and 1:1 red–blue composite light) and varying light intensities were applied for continuous illumination. The effects of these lighting conditions on the postharvest physiological quality and volatile aroma components of tomatoes were systematically investigated, and the molecular regulatory mechanisms were analyzed through transcriptome sequencing. The results showed that light quality and intensity significantly regulated tomato fruit color change, firmness, pigment accumulation, and sugar–acid quality. High-intensity red–blue combined light treatment (RB-H) effectively accelerated fruit ripening, promoted carotenoid accumulation, significantly increased the content of terpenes and aromatic volatile compounds, and improved fruit flavor. Transcriptome analysis revealed that different light treatments induced a large number of differentially expressed genes, with transcription factors from the WRKY, MYB, AP2/ERF, bHLH, NAC, and C2H2 families widely involved in light signal responses. Weighted gene co-expression network analysis (WGCNA) identified two key modules associated with light response. KEGG pathway enrichment analysis revealed that the biosynthesis of the terpenoid backbone, monoterpenoid biosynthesis, sesquiterpenoid and triterpenoid biosynthesis, as well as tyrosine metabolism pathways, are the core pathways regulating the production of volatile aroma compounds in tomatoes. In summary, RB-H treatment promotes the accumulation of characteristic aromatic compounds and enhances overall tomato quality by activating key genes involved in terpenoid biosynthesis and tyrosine metabolism pathways. This study reveals the transcriptional regulatory network by which light quality and intensity shape postharvest tomato ripening and flavor development in chitosan-oligosaccharide-pre-treated fruit, providing theoretical support for precise LED light environment control and targeted improvement of flavor quality in postharvest tomatoes.

## 1. Introduction

Tomato (*Solanum lycopersicum*) is one of the most widely cultivated and economically valuable fresh-market horticultural crops worldwide, with its commercial quality and consumer acceptance determined by fruit color development upon ripening, flesh firmness, soluble solids content, sugar-to-acid ratio, and characteristic aroma [1,2]. Regulating the ripening process of postharvest mature-green tomatoes during storage is a core research focus in improving the quality of greenhouse-grown tomatoes. Light, as a key environmental signal regulating fruit development, pigment synthesis, and flavor metabolism [3,4,5,6], has dual gradients of light quality and intensity yet to be systematically elucidated regarding their synergistic effects on tomato ripening and quality formation.

Fruit color change and pigment accumulation are the most obvious phenotypic characteristics of tomato ripening [7]. Carotenoids and anthocyanins are the key compounds responsible for the red color of tomato skin, and their biosynthetic pathways are precisely regulated by light signals [4,8,9]. Photoreceptors such as phytochromes and cryptochromes mediate downstream transcription factor cascades, directly regulating the transcriptional abundance of key structural genes involved in pigment synthesis, thereby altering fruit coloration rate and color saturation [4,5,9,10]. Existing studies have confirmed that single red and blue light can respectively promote the accumulation of carotenoids and anthocyanins in plants [10,11,12,13,14,15]. However, the differential regulatory effects of various combinations of white light, monochromatic light, and red–blue composite light at different light intensities on ripening color change and skin-color parameters of chitosan-oligosaccharide-pre-treated mature-green tomatoes remain poorly understood. Furthermore, systematic comparative research on how high- and low-intensity gradient lighting synergistically regulate the onset of color breakdown, fruit firmness, sugar–acid content, and other internal flavor qualities is still lacking.

Volatile aroma compounds form the material basis of tomato’s unique flavor, with terpenes, esters, alcohols, and heterocyclic aromatic compounds being the core components responsible for its characteristic aroma [16,17]. Among these, monoterpenes and sesquiterpenes not only contribute pleasant sensory attributes such as fruity and floral notes but are also downstream derivatives of carotenoid metabolism, whose synthesis and accumulation are simultaneously regulated by light quality signals [4,5,9,10,18]. Previous studies have primarily focused on the effect of single light qualities on total tomato aroma, lacking a comprehensive analysis of volatile metabolomes under two light intensity gradients—white light, monochromatic blue light, and 1:1 red–blue composite light. Moreover, the sensory attribute profiles associated with different aroma compounds induced by various light treatments, as well as the molecular regulatory pathways underlying differences in aromatic components between the high-flavor-quality red–blue high-intensity (RB-H) and low-intensity white light (W-L) treatments, remain unclear.

Transcriptome sequencing technology enables systematic analysis of global gene expression changes mediated by light in tomato ripening and flavor metabolism, with transcription factor families such as WRKY, MYB, AP2/ERF, bHLH, NAC, and C2H2 serving as central hubs in light signal transduction and secondary metabolic regulation [19,20,21,22,23,24]. Weighted gene co-expression network analysis (WGCNA) can identify feature gene modules highly associated with fruit ripening and aroma synthesis, and combined with KEGG pathway enrichment, it enables the identification of key pathways involved in aroma precursor biosynthesis, such as terpenoid backbone synthesis, monoterpenoid/sesquiterpenoid synthesis, and tyrosine metabolism [25,26,27]. Current studies mostly analyze the effects of light quality on the transcriptome or metabolome separately, lacking an integrated analysis of the transcription–metabolism regulatory network under multiple light gradients and chitosan oligosaccharide pre-treatment, thus failing to fully elucidate the complete molecular pathway from “light signaling to transcriptional regulation to aroma compound accumulation.”

As a natural biostimulant, chitosan oligosaccharide can enhance the stress resistance of fruits and vegetables [28,29,30], promote fruit color development, and improve flavor quality when applied as a pre-harvest spray or post-harvest dip treatment [31,32,33,34]. Nevertheless, it remains unknown how different LED supplemental light quality–intensity combinations regulate ripening and aroma metabolism in mature-green tomatoes receiving chitosan-oligosaccharide pre-treatment. This experiment used mature-green fruits of the flavor-type tomato variety “Fen Junmei” as material, pre-treated by soaking in a 0.08 g/L chitosan oligosaccharide solution. Seven treatments were established: natural indoor light control (NLS), full-spectrum white light at high and low intensities (W-H, W-L), monochromatic blue light at high and low intensities (B-H, B-L), and red–blue composite light (1:1 ratio) at high and low intensities (RB-H, RB-L). The fruits underwent seven consecutive days of stepwise supplemental lighting treatment. By observing fruit ripening phenotypes and measuring physiological quality parameters such as peel color, firmness, carotenoids, anthocyanins, soluble solids, and sugar-to-acid ratio, combined with integrated analysis of volatile metabolomics via HS-SPME-GC/MS and transcriptomics via RNA-seq, this study systematically compared the regulatory differences in postharvest tomato ripening, pigment accumulation, and flavor/aroma development under different light qualities and intensities. Key differentially expressed genes responsive to light, crucial transcription factors, and co-expression modules were identified. The expression patterns of genes involved in terpenoid biosynthesis and tyrosine metabolism were analyzed to elucidate the molecular mechanisms by which red–blue high-intensity light (RB-H) accelerates fruit ripening, enriches volatile compounds associated with fruit and floral aromas, and enhances overall fruit quality. This research provides theoretical foundations and practical technical references for precise LED light environment control and targeted improvement of postharvest flavor and quality in greenhouse tomatoes.

## 2. Results

**Figure 1:**
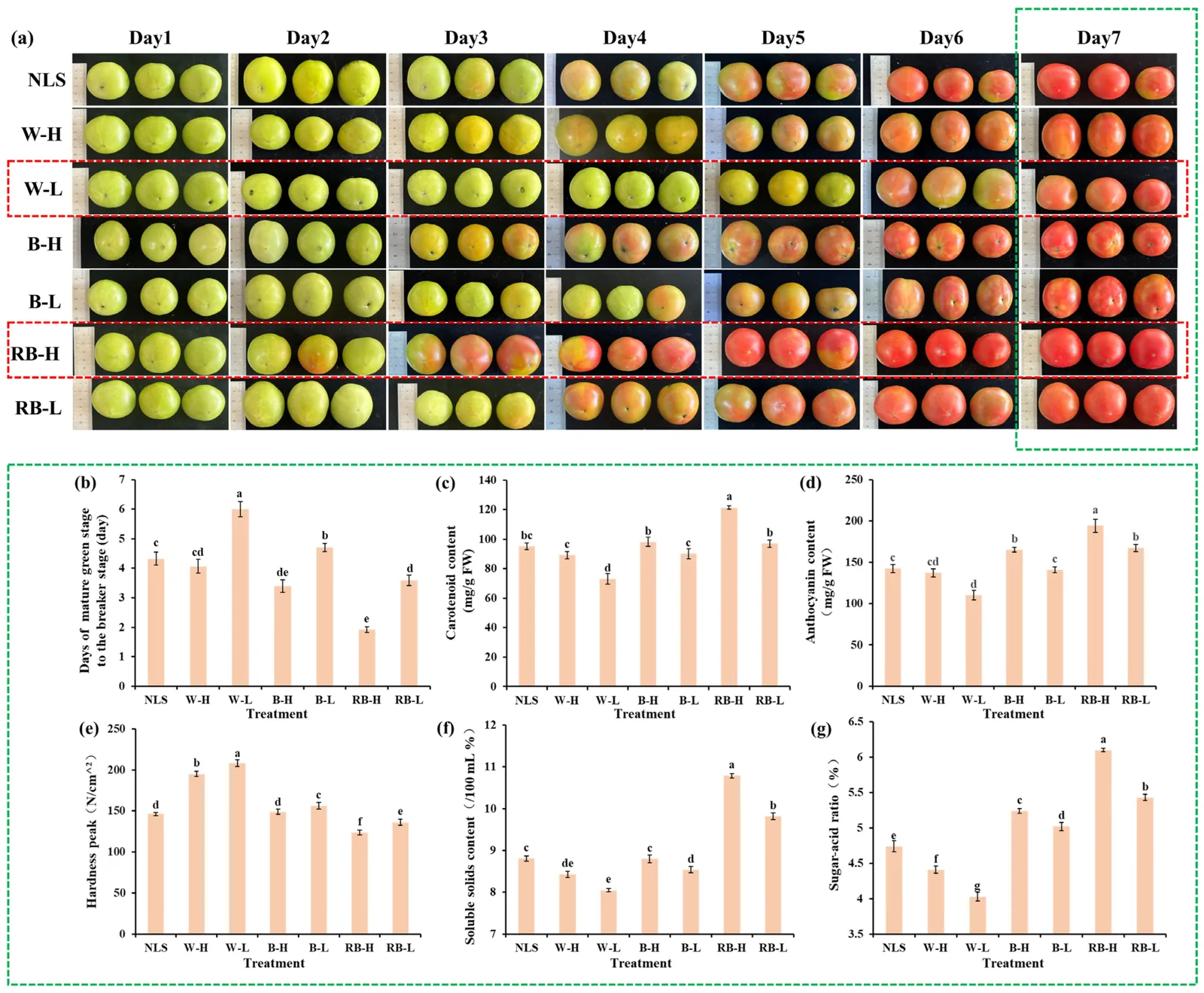
Light-regulated ripening development and physicochemical quality profiles of chitosan oligosaccharide-pre-treated green tomatoes during seven-day light incubation. (**a**) Time-series visual ripening phenotypes under natural light and various LED light quality/intensity gradients; (**b**) duration to breaker ripening stage; (**c**) accumulated carotenoids; (**d**) anthocyanin accumulation; (**e**) peak fruit firmness; (**f**) soluble solids concentration; (**g**) sugar-to-acid ratio. Different lowercase letters indicate significant separation among treatments by Duncan’s multiple range test at *p* ≤ 0.05. Vertical bars represent means ± standard error (*n* = 9).

Figure 1a intuitively shows the dynamic changes in external color development of tomato fruits under different treatments during 7 days of storage. Throughout the entire storage period, all groups gradually transitioned from green to yellow and finally developed into fully red-ripe fruits. Among them, the RB-H and W-L treatment groups, marked by dashed red and green boxes, respectively, exhibited the fastest and slowest color development among all treatments. By day 7 (indicated by the green dashed box), RB-H fruits had reached a higher degree of red ripeness, while other treatments, especially W-L, showed relatively delayed color change.

RB-H treatment significantly accelerated fruit color change and ripening, resulting in the shortest time to reach the color-break stage. It promoted the accumulation of carotenoids and anthocyanins, achieved the highest soluble solids content and sugar-to-acid ratio, and exhibited a lower peak fruit firmness with a faster softening process during ripening. In contrast, W-L treatment delayed the onset of color break, required the longest time to reach the color-break stage, suppressed pigment and soluble solids accumulation, resulted in the highest peak firmness, but had the lowest sugar-to-acid ratio. The other treatments showed ripening progress and quality parameters intermediate between RB-H and W-L, with most indicators differing significantly among treatments (Figure 1b–g).

The effects of different treatments on color parameters of tomato fruits are shown in Table 1. Regarding the red–green axis (a value), the RB-H treatment had the significantly highest a value, indicating the deepest redness, whereas the W-L treatment showed the lowest a value, reflecting the poorest red development. On the yellow–blue axis (b value), the trend was reversed: the W-L treatment had the significantly highest b value, resulting in more yellowish fruit, while the RB-H treatment had the lowest b value, indicating minimal yellow content. Chroma (C value) was highest in the RB-H treatment and lowest in the W-L treatment, suggesting higher color saturation in RB-H fruits. For hue angle (H value), RB-H had the significantly lowest H value, and W-L had the highest, further confirming that RB-H fruits were redder and W-L fruits were yellower. Lightness (L value) varied relatively little among treatments, with slightly higher values observed in W-H, B-H, and B-L treatments. Overall, the RB-H treatment significantly promoted red development and enhanced color saturation in tomato fruits, while the W-L treatment delayed fruit reddening and resulted in a more yellowish skin.

**Figure 2:**
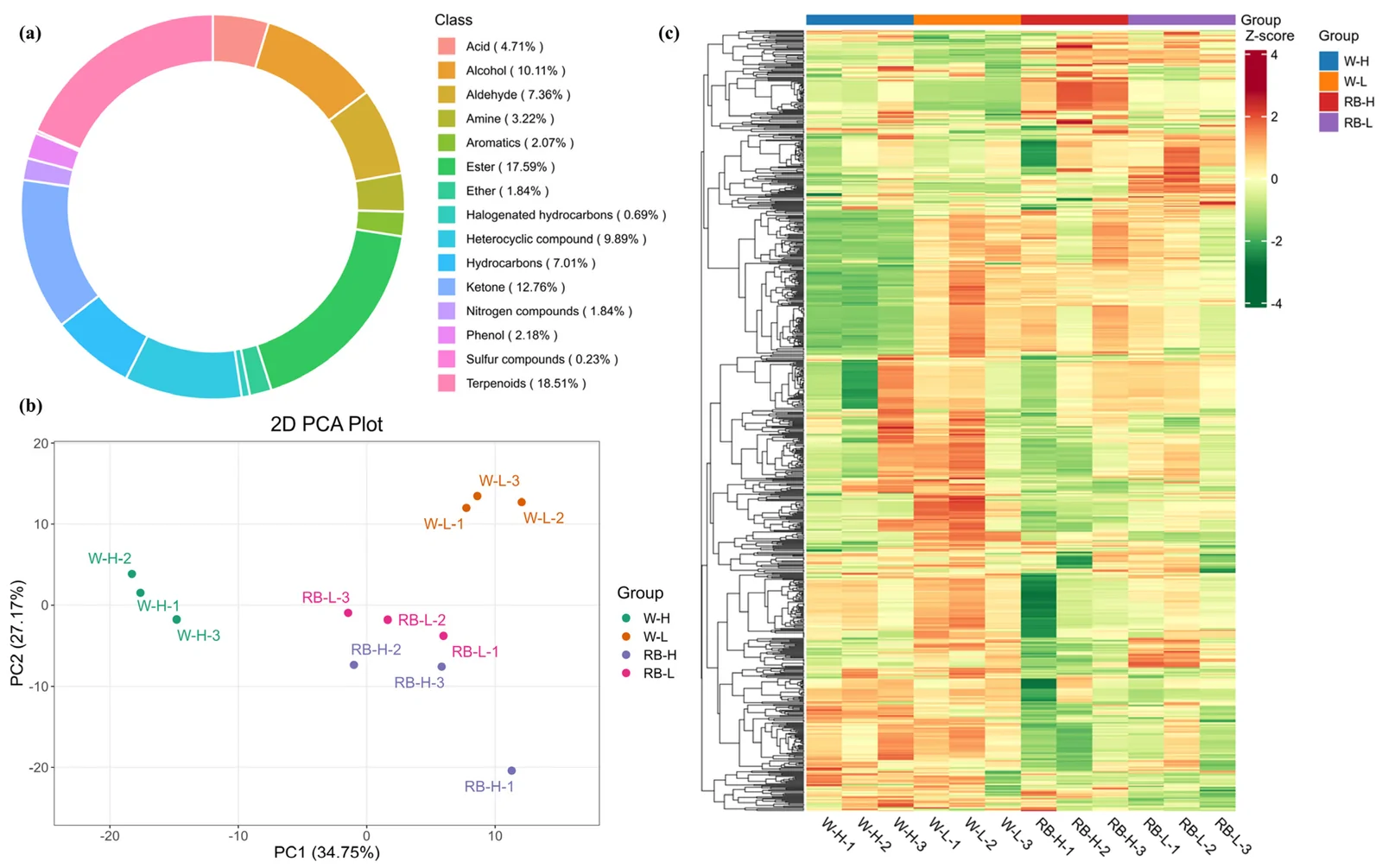
Light-quality and intensity-dependent variation in volatile organic compound metabolomes of postharvest green tomatoes after seven days of illumination. (**a**) Metabolite Class Count Ring, classification and proportion of total 870 volatile organic compounds (VOCs) detected in tomato fruits; (**b**) principal component analysis (PCA) ordination demonstrating distinct clustering patterns of fruit VOC metabolomes from W-H, W-L, RB-H and RB-L light treatments; (**c**) hierarchical clustering heat map of VOC accumulation in tomato fruits.

As shown in Figure 2a, the volatile compounds detected in this study can be classified into 12 major categories, among which terpenoids (18.51%), esters (17.59%), ketones (12.76%), alcohols (10.11%), and heterocyclic compounds (9.89%) are the primary volatile components, collectively forming the foundation of tomato’s characteristic aroma. Principal component analysis was performed based on all volatile compounds (Figure 2b), with PC1 and PC2 together accounting for 61.92% of the total sample variance. The samples from each group showed distinct separation in the two-dimensional space: W-H group samples were concentrated in the negative region of PC1, W-L group clustered in the positive regions of both PC1 and PC2, while RB-H and RB-L samples were mainly distributed in the right-central area. The samples within each group exhibited strong cohesion, with clear boundaries between groups, indicating that different treatments significantly altered the overall volatile compound profiles of tomato fruits. The volatile compound clustering heatmap (Figure 2c) further confirms the aforementioned differences. After Z-score normalization, distinct accumulation patterns of various volatile compounds across the four treatments are evident, with numerous treatment-specific high-abundance compounds observed. Significant upregulation and downregulation of volatile compounds among different treatments are apparent, and each group—W-H, W-L, RB-H, and RB-L—exhibits a unique profile of aroma compound accumulation, indicating that treatment conditions can regulate the synthesis and accumulation of tomato volatile components.

**Figure 3:**
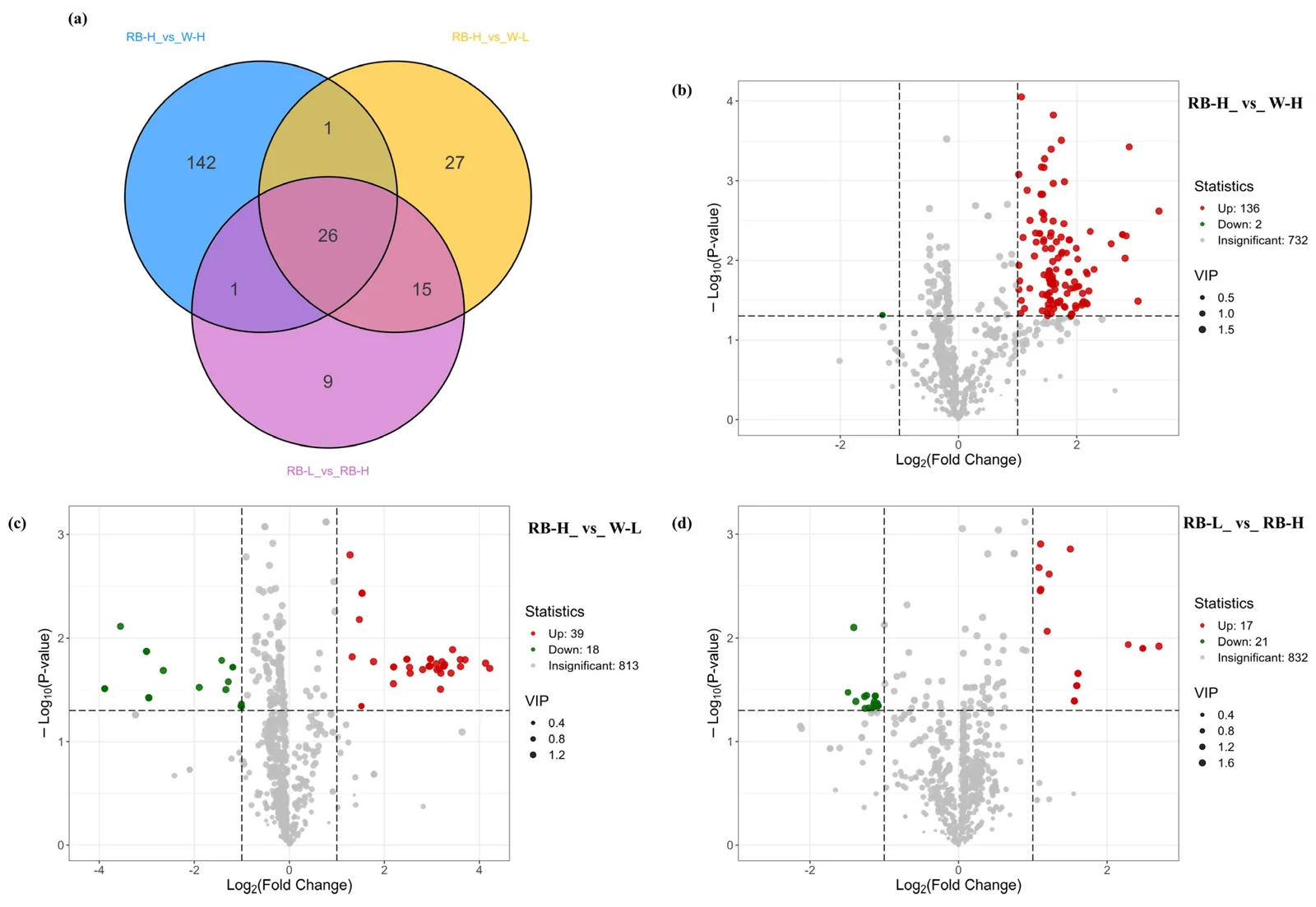
Differential volatile organic compounds (DVOCs) between different comparison groups of postharvest green tomatoes after seven days of illumination. (**a**) Venn diagram showing the overlap of DVOCs identified from RB-H_vs_W-H, RB-H_vs_W-L and RB-L_vs_RB-H comparisons; (**b**–**d**) volcano plots illustrating the overall distribution of DEMs in RB-H_vs_W-H (**b**), RB-II_vs_W-L (**c**) and RB-L_vs_RB-H (**d**); red dots represent significantly up-regulated VOCs, green dots represent significantly downregulated VOCs, and gray dots denote VOCs with non-significant expression changes, with dot size corresponding to variable importance in projection (VIP) values.

Based on pairwise comparisons among multiple groups, the Venn diagram results (Figure 3a) revealed a large number of unique and shared differentially volatile compounds across the three comparisons: RB-H vs. W-H, RB-H vs. W-L, and RB-L vs. RB-H. Among them, RB-H showed the highest number of unique differentially expressed compounds when compared to W-H (142 types). A total of 26 volatile compounds were commonly identified across all three groups, representing core aromatic components with stable responses among different treatments. Each group also contained varying numbers of unique differentially expressed metabolites, indicating that distinct treatments can induce specific accumulation of volatile compounds.

The volcano plot clearly illustrates the distribution patterns of upregulated and downregulated volatile compounds across different groups (Figure 3b–d). Comparing RB-H vs. W-H, a total of 138 differentially expressed volatile compounds were identified, including 136 significantly upregulated and 2 significantly downregulated compounds; RB-H vs. W-L yielded 57 differentially expressed compounds, with 39 upregulated and 18 downregulated; RB-L vs. RB-H showed 38 differentially expressed compounds, comprising 17 upregulated and 21 downregulated. The comparison reveals that RB-H treatment induced a substantial increase in the expression of volatile compounds compared to white light treatments (W-H, W-L), whereas between RB-L and RB-H, the numbers of upregulated and downregulated compounds were relatively balanced, resulting in smaller differences in aromatic components between these two groups.

**Figure 4:**
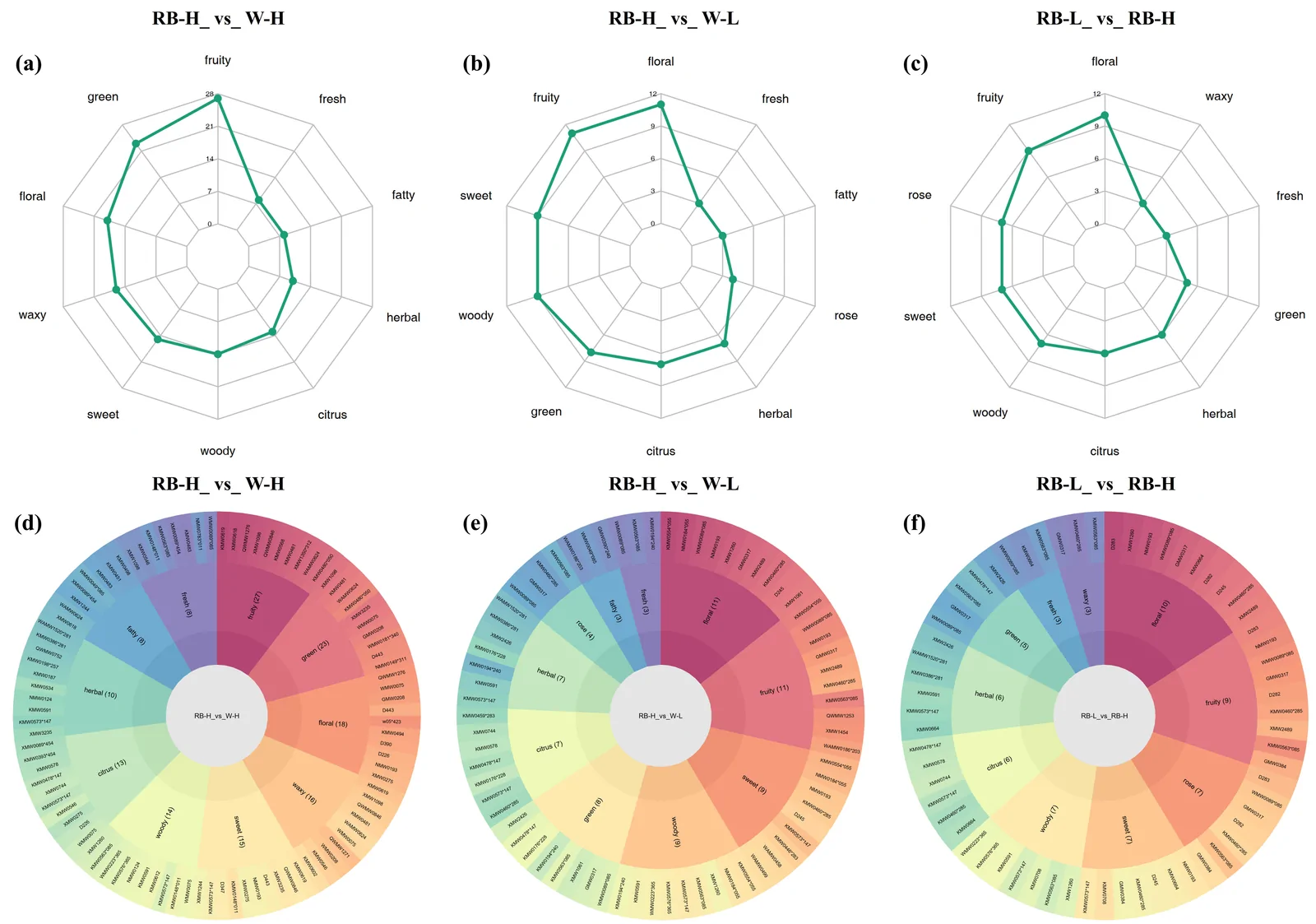
Sensory attribute distribution and classification of DVOCs in three pairwise comparison groups. (**a**–**c**) Flavor radar charts showing the quantitative scores of ten aroma sensory attributes for comparisons of RB-H_vs_W-H (**a**), RB-H_vs_W-L (**b**), and RB-L_vs_RB-H (**c**); the labels on the outermost circle denote sensory flavor profiles, and the numbers corresponding to the green dots represent the occurrence count of each sensory flavor profile—specifically, the number of DVOCs annotated to that particular sensory flavor feature. (**d**–**f**) Flavor sunburst diagrams illustrating the category annotation of DVOCs corresponding to RB-H_vs_W-H (**d**), RB-H_vs_W-L (**e**), and RB-L_vs_RB-H (**f**); the innermost circle denotes the differential comparison group; the middle circle presents the top 10 sensory flavor attributes most frequently annotated to the DVOCs within that group, with parenthetical numerals indicating the count of DVOCs linked to each attribute; and the outermost circle lists the corresponding differential metabolites—specifically, when an attribute is associated with more than 10 DVOCs, only those with the top 10 VIP values are displayed.

The sensory characteristics of aroma were analyzed based on volatile compounds, and radar charts clearly illustrated the differences in aroma profiles among groups (Figure 4a–c). In the RB-H vs. W-H comparison, compounds associated with fruity, green, floral, waxy, and sweet aromas showed prominent contributions. In the RB-H vs. W-L group, higher scores were observed for fruity, sweet, woody, green, and floral attributes. The aroma profile of the RB-L vs. RB-H group exhibited overall lower intensity compared to the first two groups, with fruity, floral, rose, and sweet notes as the main characteristic aromas. Overall, the RB-H treatment significantly enhanced the levels of volatile compounds related to pleasant aromas such as fruity, floral, and sweet compared to white light treatments (W-H, W-L).

The circular classification diagram of volatile aroma compounds (Figure 4d–f) further quantifies the composition of different aroma groups. Compared with W-H and W-L, RB-H showed a higher abundance of fruity, green, and floral aroma compounds; whereas the differences in aroma compound levels between RB-L and RB-H were relatively mild. The results indicate that different treatments reshape the aroma profile of tomato fruits by modulating the accumulation of volatile compounds with distinct sensory attributes, with RB-H being more effective in enriching characteristic fruity and floral aroma compounds, thereby improving tomato flavor quality. Based on the above data, we clarify that sensory profiles are inferred from chemically identified volatile metabolites linked to documented flavor descriptors, rather than from human panel evaluation.

**Figure 5:**
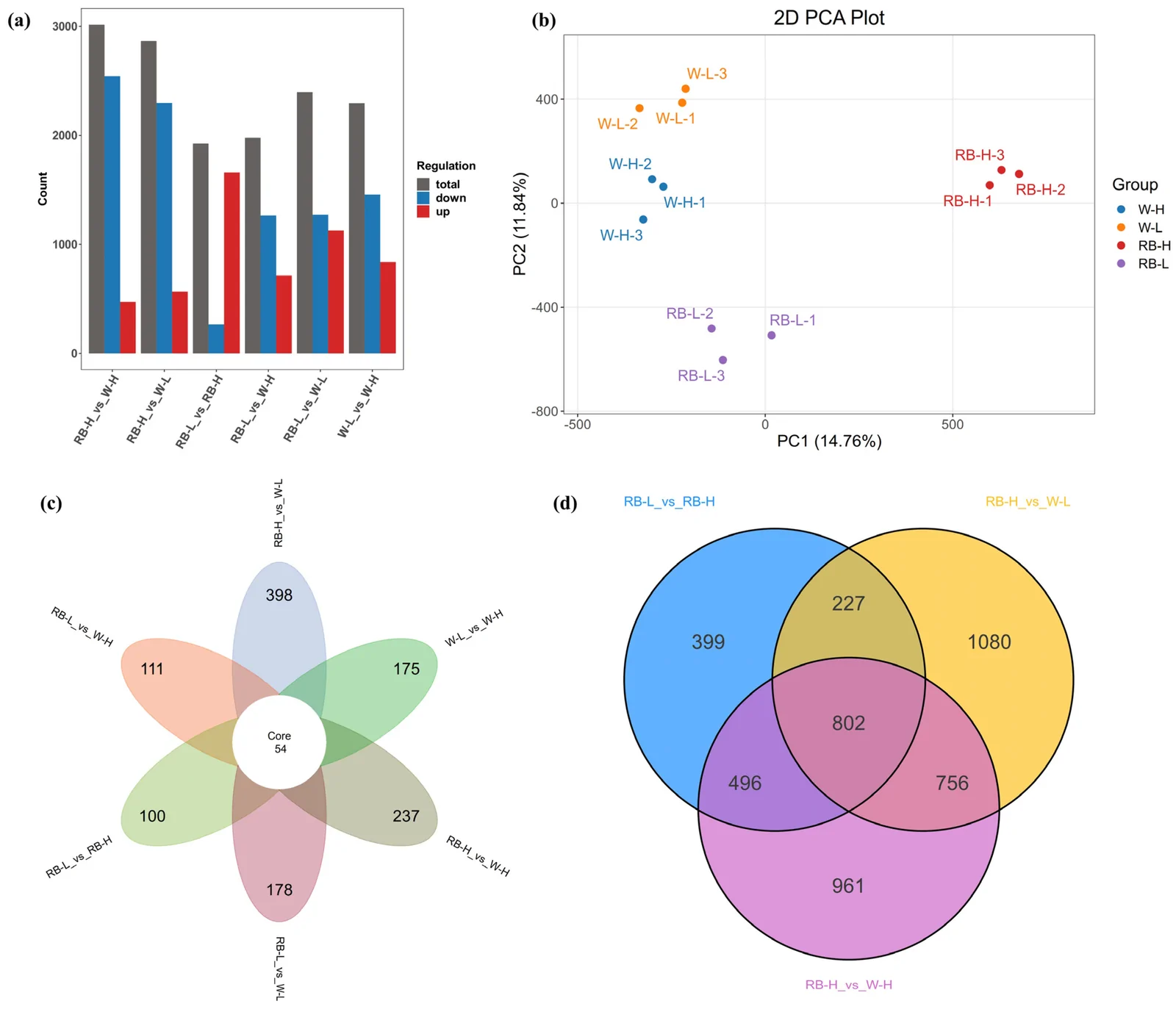
Global overview of differentially expressed genes (DEGs) across all pairwise comparison groups. (**a**) Bar chart displaying the total number, upregulated and downregulated differential features in each comparison group; gray bars = total features, red bars = significantly upregulated features, blue bars = significantly downregulated features. (**b**) Two-dimensional principal component analysis (2D PCA) plot based on all detected features, showing the separation of four sample groups (W-H, W-L, RB-H, RB-L); PC1 and PC2 represent the top two principal components with corresponding explained variance labeled on axes. (**c**) Flower Venn diagram presenting shared and unique differential features among all six pairwise comparison groups, with the central core representing common differential features existing in all comparisons. (**d**) Triple Venn diagram illustrating the overlap of differential features derived from three key comparison groups: RB-H_vs_W-H, RB-H_vs_W-L and RB-L_vs_RB-H.

To analyze the effects of different light quality treatments on gene transcription levels in tomato fruit, global expression profiles and differentially expressed genes were examined based on transcriptome data. The bar chart showing the count of differentially expressed genes (Figure 5a) revealed a large number of differentially expressed genes across all comparisons. In the comparisons between RB-H vs. W-H and RB-H vs. W-L, the number of differentially expressed genes was high, with significantly more downregulated genes than upregulated ones. In contrast, the proportion of upregulated genes increased markedly in the comparisons RB-L vs. RB-H, RB-L vs. W-H, and RB-L vs. W-L, indicating distinct transcriptional regulatory patterns between RB-H and white light treatments (W-H, W-L), as well as between RB-H and RB-L. Detailed statistics are provided in Table S1. PCA results (Figure 5b) showed that the four groups—W-H, W-L, RB-H, and RB-L—were clearly separated in the two-dimensional space, with biological replicates within each treatment tightly clustered. This indicates that different light quality conditions significantly reshape the gene expression profiles of tomato fruits, and the sequencing data exhibit good reproducibility, confirming reliable and authentic differences in gene expression among groups.

Venn diagrams further elucidate the shared and unique relationships among differentially expressed genes. The petal Venn diagram (Figure 5c) reveals that there are 54 core common differentially expressed genes across all five comparisons, representing conserved key genes involved in tomato’s response to light changes. Meanwhile, each group contains a varying number of unique differentially expressed genes, reflecting specific transcriptional responses induced by different light combinations. Focusing on the three comparisons related to RB-H (Figure 5d), the three groups shared 802 differentially expressed genes; RB-H vs. W-L had the highest number of unique differentially expressed genes (1080), followed by RB-H vs. W-H with 961, and RB-L vs. RB-H with 399. These unique differentially expressed genes could serve as candidate targets for elucidating how red–blue composite light and white light, as well as different red-to-blue light ratios, regulate tomato ripening and flavor development.

**Figure 6:**
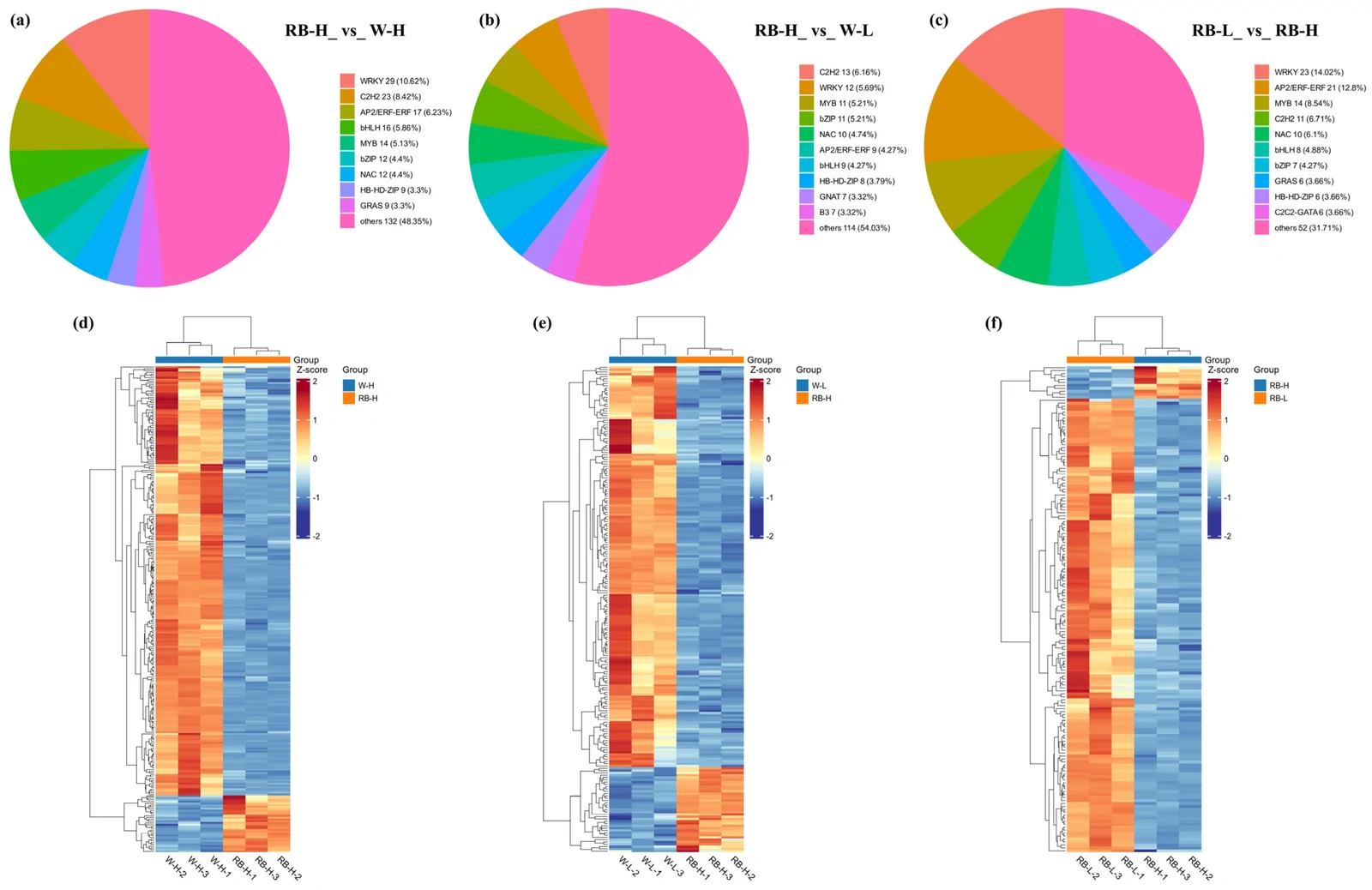
Classification and expression clustering of differentially expressed transcription factors (DETFs) in three pairwise comparison groups. (**a**–**c**) Pie charts showing the proportion of transcription factor (TF) families among DETFs identified from RB-H_vs_W-H (**a**), RB-H_vs_W-L (**b**), and RB-L_vs_RB-H (**c**); each colored sector represents one TF family, with percentages indicating the proportion of corresponding DETFs, and the pink sector denotes all remaining minor TF families grouped as “others”. (**d**–**f**) Hierarchical clustering heatmaps of all DETFs for the comparison groups RB-H_vs_W-H (**d**), RB-H_vs_W-L (**e**), and RB-L_vs_RB-H (**f**), respectively. The color gradient represents normalized Z-score of gene expression, where red indicates high expression and blue indicates low expression; rows correspond to individual DETFs with clustering dendrogram on the left, columns represent biological replicates of each sample group.

Figure 6 shows the statistical analysis and expression pattern of differentially expressed transcription factor families. Pie chart results indicate that WRKY, MYB, AP2/ERF, bHLH, NAC, and C2H2 are core transcription factor families significantly enriched across all three comparisons (Figure 6a–c). Clustering heatmaps reveal that these differentially expressed transcription factors exhibit distinct treatment-specific expression patterns (Figure 6d–f): RB-H shows significant differences in transcription factor expression profiles compared to white light treatments (W-H, W-L), and there is also clear divergence in transcription factor expression between RB-H and RB-L groups. This suggests that light quality and intensity participate in the transcriptional regulation of tomato fruit ripening and volatile compound biosynthesis by modulating the expression of numerous transcription factors. Detailed statistics on differentially expressed transcription factors among the comparisons RB-H vs. W-H, RB-H vs. W-L, and RB-L vs. RB-H are provided in Table S2.

**Figure 7:**
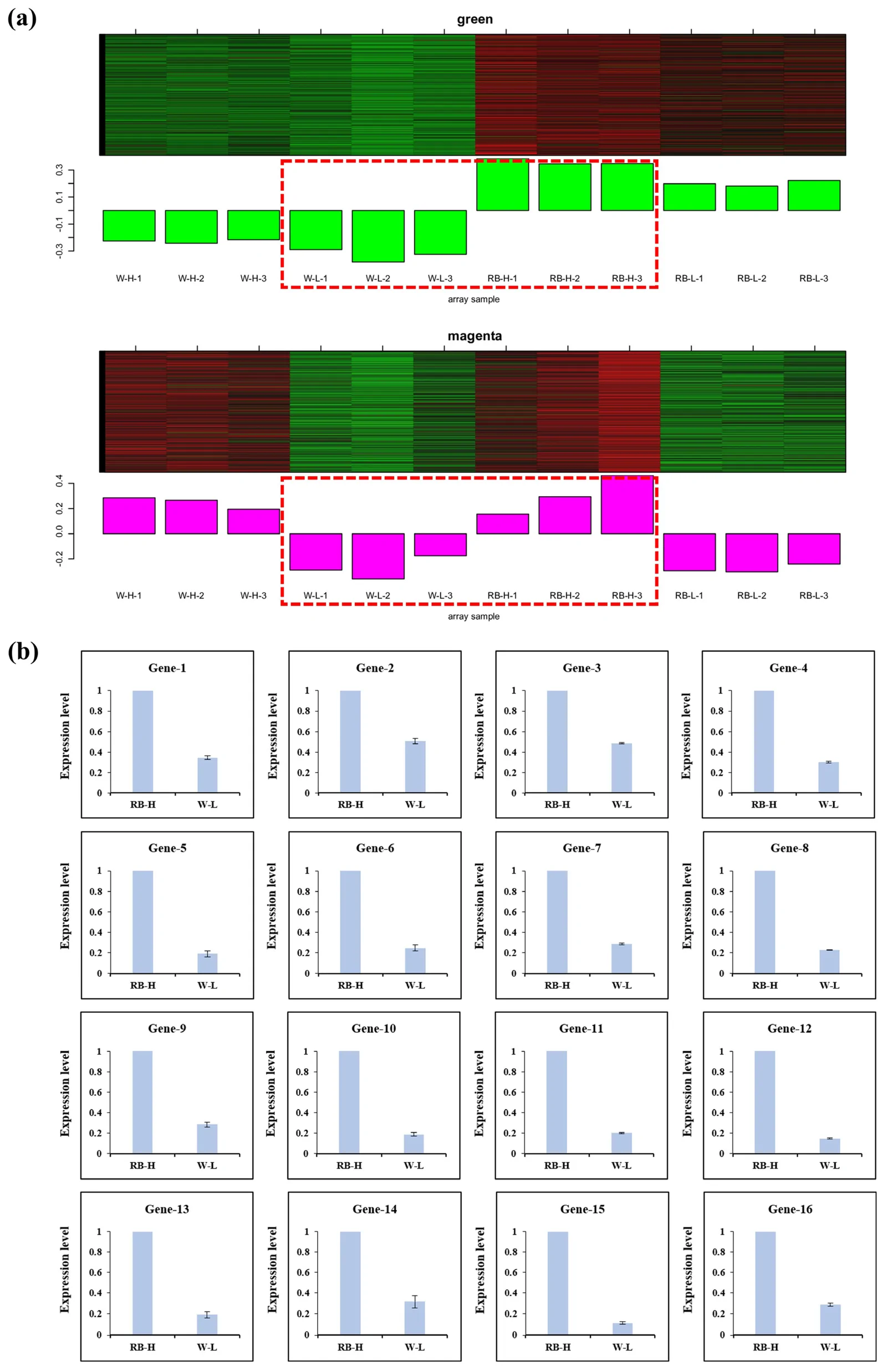
WGCNA module expression patterns and validation of representative hub genes between RB-H and W-L groups. (**a**) Heatmaps and eigengene expression bar plots of the green and magenta WGCNA modules. The upper heatmap of each module shows normalized gene expression profiles across all samples (green = low expression, red = high expression); the lower bar plot displays module eigengene values for each biological replicate, with the red dashed rectangle highlighting the sample set of W-L and RB-H for subsequent gene verification. (**b**) Bar charts showing the relative expression levels of 16 representative hub genes selected from the target modules in RB-H versus W-L groups; vertical bars represent means ± standard error (*n* = 3).

Weighted gene co-expression network analysis (WGCNA) was employed to identify core gene modules associated with light treatment responses (Figure 7a, Table S3). Two key modules—green and magenta—were selected. Genes in the green module showed generally low expression in W-H and W-L groups but were significantly upregulated under RB-H and RB-L treatments. Similarly, genes in the magenta module exhibited distinct group-specific expression patterns, with suppressed expression in the W-L group and significant enrichment in the RB-H group. The expression trends of module eigengenes clearly confirmed the differential expression between these two modules, suggesting that genes within the green and magenta modules may mediate tomato responses to different light qualities and intensities, likely regulating fruit ripening and flavor metabolism. A total of 16 core candidate genes from these two modules were selected for further validation of their expression profiles (Figure 7b). The results showed that the expression levels of all candidate genes were significantly higher in the RB-H group compared to the W-L group, fully consistent with the expression trends of the WGCNA modules. These candidate genes can serve as key targets for further investigation into light-quality- and-intensity-dependent regulation of tomato aroma-compound biosynthesis pathways.

**Figure 8:**
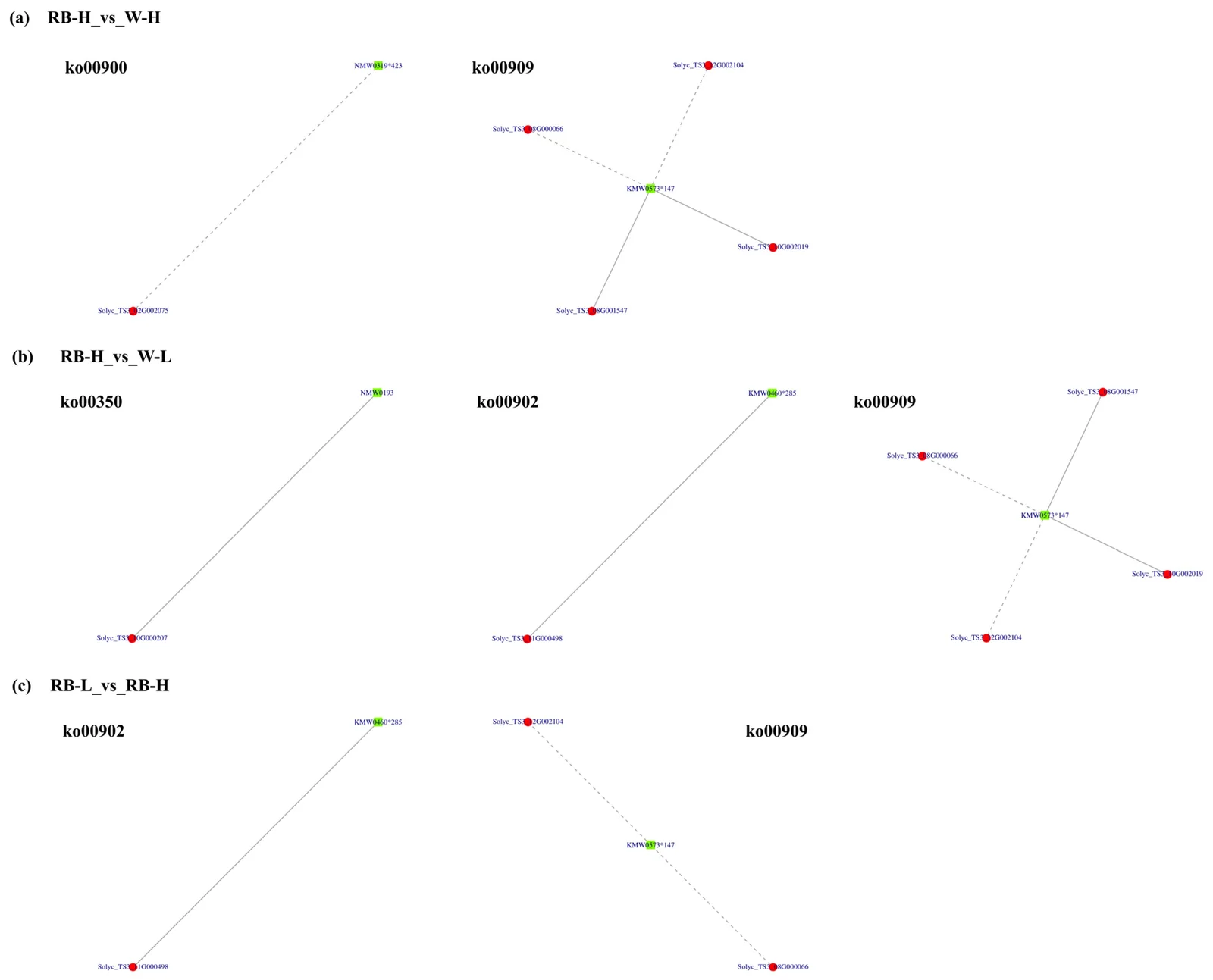
KEGG interaction networks of key differential genes enriched in significantly altered pathways across three pairwise comparison groups. (**a**) Gene connection networks of enriched KEGG pathways ko00900 and ko00909 in the RB-H_vs_W-H comparison group; (**b**) gene interaction diagrams of KEGG pathways ko00350, ko00902 and ko009029 for the RB-H_vs_W-L comparison group; (**c**) gene linkage networks of KEGG pathways ko00902 and ko00909 derived from the RB-L_vs_RB-H comparison group. Red nodes represent significantly upregulated genes, green nodes represent core pathway intermediate genes; solid lines indicate direct functional interactions between genes, and dashed lines represent indirect regulatory relationships.

Figure 8 shows the pathway gene interaction network constructed from KEGG enrichment results of differentially expressed genes, revealing the collaborative relationships among genes within key metabolic pathways under light regulation (Table S4). The sesquiterpenoid and triterpenoid biosynthesis pathway (ko00909) showed significant gene interactions in all three comparisons—RB-H vs. W-H, RB-H vs. W-L, and RB-L vs. RB-H—making it a core responsive pathway across treatments. Additionally, the terpenoid backbone biosynthesis pathway (ko00900) was uniquely enriched in the RB-H vs. W-H comparison; the tyrosine metabolism pathway (ko00350) and monoterpenoid biosynthesis pathway (ko00902) were additionally enriched in RB-H vs. W-L; while the monoterpenoid biosynthesis pathway (ko00902) was enriched in RB-L vs. RB-H. In the pathway network, numerous upregulated genes were associated with central hub genes of the pathways, indicating that different light qualities alter tomato fruit metabolism by activating multiple metabolic pathways and coordinating gene regulation within these pathways.

**Figure 9:**
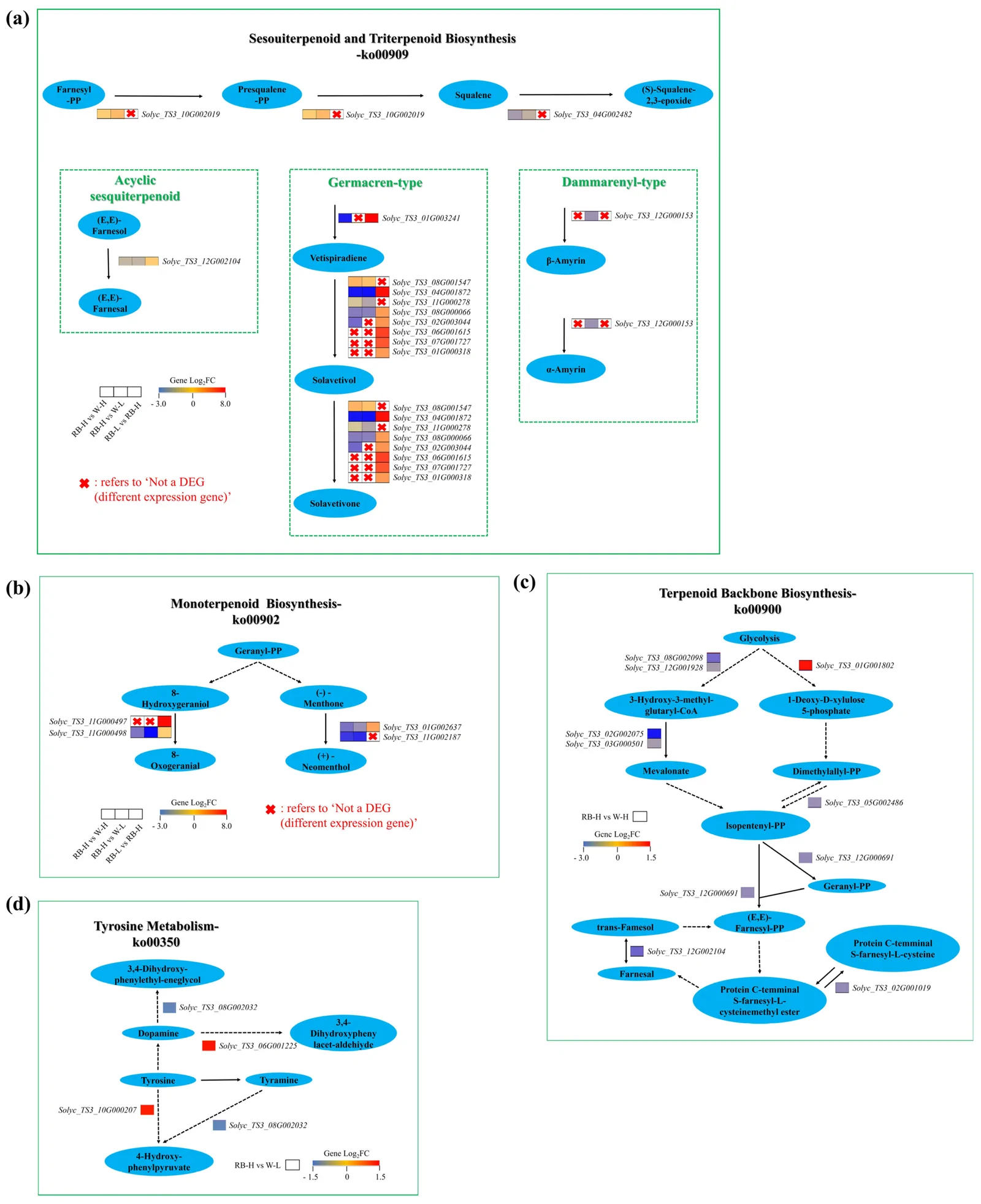
Schematic diagrams of four key enriched KEGG pathways with differential gene expression annotation across three pairwise comparison groups. (**a**) Sesquiterpenoid and triterpenoid biosynthesis pathway (ko00909); (**b**) monoterpenoid biosynthesis pathway (ko00902); (**c**) terpenoid backbone biosynthesis pathway (ko00900); (**d**) tyrosine metabolism pathway (ko000350). Blue ovals represent intermediate metabolites in each pathway; colored rectangular heatmaps beside enzyme gene IDs show the log_2_ (Fold Change) values of corresponding genes in RB-H_vs_W-H, RB-H_vs_W-L and RB-L_vs_RB-H groups; red cross marks indicate genes without significant differential expression (non-DEGs); the color gradient scale at the bottom left of each panel illustrates the range of gene log_2_ (Fold Change).

Based on the KEGG enrichment results of differentially expressed genes, we focused on analyzing changes in gene expression within the terpenoid biosynthesis and tyrosine metabolism pathways (Figure 9). Terpenoids are important precursors of volatile aromatic compounds in tomatoes. The three terpenoid biosynthesis pathways—terpenoid backbone biosynthesis (ko00900), monoterpenoid biosynthesis (ko00902), and sesquiterpenoid and triterpenoid biosynthesis (ko00909)—were all significantly enriched.

In the sesquiterpenoid and triterpenoid biosynthesis pathway (ko00909), significant differences were observed in multiple downstream branching pathway genes following farnesyl pyrophosphate. Genes associated with germacrene-type synthesis showed a clear upregulation trend, while genes involved in acyclic sesquiterpenoids and dammarane-type synthesis also exhibited differential expression, indicating that RB-H treatment promotes the synthesis of sesquiterpene aroma compounds (Figure 9a). In the monoterpenoid biosynthesis pathway (ko00902), key genes responsible for producing downstream compounds such as neomenthol and 8-hydroxygeraniol from geranyl diphosphate were significantly upregulated in the RB-H group compared to the white light group, favoring the accumulation of monoterpene flavor compounds (Figure 9b). The terpenoid backbone pathway (ko00900), as an upstream pathway, undergoes altered expression of multiple key enzyme-encoding genes, providing the foundational precursor supply for downstream monoterpenoid and sesquiterpenoid biosynthesis (Figure 9c). The tyrosine metabolism pathway (ko00350) is closely associated with the production of aromatic volatile compounds (Figure 9d). Genes involved in the conversion steps of tyrosine, tyramine, and dopamine within this pathway were significantly upregulated in the RB-H group compared to the W-L group, promoting the synthesis of aromatic aroma compounds.

Overall, compared to W-H and W-L white light treatments, RB-H can simultaneously activate a large number of key genes in both the terpenoid biosynthesis pathway and the tyrosine metabolism pathway, promoting the production of monoterpenes, sesquiterpenes, and aromatic volatile compounds, whereas RB-L shows reduced differences in pathway gene expression relative to RB-H. This result provides a molecular explanation at the transcriptional level for the differences in volatile aroma components in tomatoes under different light quality treatments.

## 3. Discussion

### 3.1. Regulation of Postharvest Tomato Ripening and External Coloration by Light Quality and Intensity in Chitosan-Oligosaccharide-Pre-Treated Fruit

Fruit color change, skin color, and firmness are key indicators for evaluating the postharvest ripening process and commercial appearance quality of tomatoes [35,36]. Light signals can regulate chlorophyll degradation and carotenoid and anthocyanin biosynthesis pathways through downstream transcriptional cascades mediated by photoreceptors [4,5,6,10]. Chitosan oligosaccharides, as biostimulants, can activate endogenous ripening-related metabolic pathways in fruits and vegetables [28,29,31,32,33,34]. The primary scientific question addressed here is how light signals modulate ripening in chitosan-oligosaccharide-pre-treated mature-green tomatoes. The results of this study (Figure 1 and Table 1) indicate that high-intensity 1:1 red–blue composite light treatment (RB-H) significantly shortened the time for mature-green tomatoes to reach the color-break stage, accelerated chlorophyll degradation, substantially increased the fruit skin a* value and chroma C, decreased the hue angle H°, and reduced fruit firmness. In contrast, low-intensity white light (W-L) significantly delayed fruit color change, suppressed pigment accumulation, and maintained higher flesh firmness—consistent with previous findings that red light and blue light individually promote carotenoid and anthocyanin accumulation in tomatoes or other plants [37,38,39], respectively. Compared to monochromatic light and full-spectrum white light, red–blue composite light achieves an additive effect by combining red and blue light signals [40,41,42]. Red light promotes ethylene synthesis via the photoreceptor PHY [43,44,45], accelerating fruit ripening, while blue light upregulates key carotenoid biosynthesis enzyme genes through cryptochrome CRY [46,47]. High light intensity further amplifies the strength of light signal transduction [48,49,50,51]. These light-driven physiological changes take place in fruits that had received uniform 0.08 g/L chitosan-oligosaccharide pre-treatment, which is known to soften cell walls and favor pigment synthesis in fruit tissues (Figure 1 and Table 1).

This study found that the regulatory differences caused by light intensity gradients are consistently present under the same light quality: within the same light quality system, high-intensity treatments showed superior color change rates, pigment content, and sugar–acid quality compared to low-intensity treatments (Figure 1 and Table 1), indicating a dose-dependent effect of light signals, where sufficient photon flux can fully activate downstream ripening-related gene expression [52,53]. However, the regulatory effect of light quality takes precedence over light intensity; for example, RB-L (red–blue low intensity) still outperformed W-H (white light high intensity) across all quality parameters, demonstrating that the red–blue spectral combination is the key light environment condition for enhancing postharvest ripening quality in tomatoes. The natural light control group (NLS) had a light intensity of only 50 μmol m^−2^ s^−1^, with a spectral composition lacking sufficient red and blue light—likely the key reason for its inability to effectively drive pigment and flavor metabolism [54]. Its overall quality was inferior to all artificial LED supplemental lighting treatments, confirming that postharvest targeted LED supplementation is an effective technique for improving the postharvest quality of green-ripe tomatoes.

### 3.2. Differential Regulation of Sugar–Acid Flavor and Volatile Aroma Metabolism in Tomatoes Under Different Light Environments

The sugar-to-acid ratio directly determines the basic palatability of tomatoes [1,2,3], while terpenoids and aromatic volatile compounds contribute to the characteristic fruity and floral flavors [16,17,18]. In this study, the RB-H treatment group exhibited the highest levels of soluble solids and sugar-to-acid ratio among all treatments, whereas the W-L group showed the lowest (Figure 1). This difference arises from the regulation by light signals on the transport of photosynthetic products and organic acid degradation pathways: high-intensity red–blue composite light upregulates fruit sucrose transporter genes, promoting the conversion of starch into soluble sugars [55,56,57,58,59], while simultaneously activating enzymes involved in organic acid degradation [60], reducing citric acid accumulation, and thereby optimizing the sugar-to-acid balance.

A total of 870 volatile compounds in tomatoes were detected using HS-SPME-GC/MS, with terpenes being the dominant aromatic component, consistent with previous fundamental research on tomato aroma metabolism (Figure 2a). Pairwise analysis of differential volatile compounds revealed that RB-H treatment significantly upregulated over a hundred aroma compounds compared to white light treatment (Figure 3b–d), and was enriched with numerous fruit-, flower-, and sweet-scented terpenes and aromatic metabolites, resulting in a significantly superior sensory profile compared to white light treatment. In contrast, the W-L group showed the lowest overall accumulation of aroma compounds, with an increased proportion of negative flavors such as green grassy notes (Figure 4). Pathway enrichment analysis confirmed that terpenoid backbone biosynthesis, monoterpenoid synthesis, sesquiterpenoid and triterpenoid synthesis, and tyrosine metabolism are the core pathways regulating light-mediated aroma formation (Figure 8 and Figure 9). The upstream terpenoid pathway provides isopentenyl diphosphate precursors for downstream monoterpene and sesquiterpene synthesis [18]. RB-H treatment may simultaneously activate key rate-limiting enzyme genes such as *DXS* and *HMGR* within this pathway, leading to abundant production of geranyl diphosphate and farnesyl diphosphate, which, in turn, synthesize characteristic fruit aromas like limonene and β-farnesene [61,62,63,64]. Meanwhile, the tyrosine metabolism pathway regulates the biosynthesis of phenolic and aromatic heterocyclic aroma compounds [65,66,67]. This pathway showed significant enrichment only in the comparison between RB-H and W-L, explaining the molecular mechanism behind the notable accumulation of aromatic compounds under red–blue high-intensity light treatment.

Notably, although low-intensity red–blue light treatment (RB-L) also upregulates genes in the terpenoid pathway, the degree of upregulation is significantly lower than that of RB-H (Figure 9), resulting in a marked difference in the accumulation of aromatic compounds. This indicates that light intensity can achieve dose-dependent regulation of aroma components by modulating the transcriptional levels of metabolic pathways [68]. In contrast, the white light system may lack the red-light-mediated signal for secondary metabolite activation [69,70,71]; even under high-intensity white light (W-H), characteristic terpenoid aromas cannot be synthesized in large quantities. Thus, the spectral composition of light quality is a decisive factor determining the flavor quality of tomatoes.

### 3.3. Transcription Factors and Co-Expressed Modules Mediate Light Signal Regulation of Tomato Ripening and Aroma Metabolism

Transcriptome analysis identified six major differentially expressed transcription factor families—WRKY, MYB, AP2/ERF, bHLH, NAC, and C2H2—as key hubs linking light signaling to downstream pathways involved in ripening and aroma metabolism (Figure 6). MYB transcription factors have been shown to directly bind to promoters of carotenoid and anthocyanin biosynthesis, regulating pigment accumulation [72,73,74]; NAC and AP2/ERF families are core regulators in the ethylene signaling pathway [21,75,76], mediating fruit ripening and softening [77,78,79]; WRKY and bHLH can simultaneously regulate secondary metabolism and light signal responses [80,81,82,83,84]. In this study, the aforementioned transcription factors were specifically highly expressed in the RB-H group, synergistically activating pigment and terpenoid biosynthesis pathways, whereas their overall expression was suppressed in the W-L group, leading to a concurrent decline in metabolic pathway activity (Figure 6a–c and Table S2). The transcription factor expression profiles under different treatments were completely distinct (Figure 6d–f), demonstrating that light quality and intensity achieve differential regulation of ripening and flavor metabolism by reshaping the transcription factor regulatory network.

WGCNA-based weighted gene co-expression network analysis identified two light-responsive core modules, green and magenta. Genes in the green module were significantly upregulated under red and blue mixed light treatments, while genes in the magenta module showed specific high expression levels in the high-light intensity groups (W-H and RB-H) (Figure 7a). Among the 16 core hub genes within the module, all were confirmed by qRT-PCR to be significantly upregulated in the RB-H group (Figure 7b). Module function annotations revealed that the two major modules were enriched with numerous structural genes and transcription factors related to amino acid metabolism, terpenoid biosynthesis, flavonoid synthesis, fatty acid synthesis, and ethylene biosynthesis (Table S3), forming a complete regulatory unit of “light signaling–transcriptional regulation–metabolite accumulation.” In contrast, genes in the W-L group module were generally silenced, leading to blocked metabolic pathways, thus explaining at the global transcriptional level the molecular mechanism by which low-intensity white light suppresses tomato quality formation. The 54 core differentially expressed genes shared across all five comparisons can serve as conserved marker genes for tomato light response (Figure 5a), providing candidate targets for future functional validation of genes involved in tomato light signaling pathways.

### 3.4. Multi-Omics Integration to Elucidate the Molecular Network of Light-Regulated Tomato Aroma Metabolic Pathways

Transcriptome-volatile metabolome association analysis revealed that four pathways—terpenoid backbone biosynthesis (ko00900), monoterpenoid biosynthesis (ko00902), sesquiterpenoid and triterpenoid biosynthesis (ko00909), and tyrosine metabolism (ko00350)—are the core pathways through which RB-H improves tomato flavor. Numerous structural genes within these pathways showed highly significant positive correlations with differentially volatile terpenoid and aromatic compounds (Figure 8 and Figure 9, Table S4). In the sesquiterpene pathway, genes involved in the synthesis of germacrene-type compounds were significantly upregulated in RB-H, leading to a notable enrichment of fruit-aroma compounds such as germacrene and farnesene. In the monoterpene pathway, key enzymes for the synthesis of neomenthol and geraniol showed high expression, increasing the levels of floral monoterpenes [85,86]. Meanwhile, the tyrosine metabolic pathway promoted the production of aromatic compounds derived from dopamine and tyramine [87,88], enriching the complex aroma profile of tomatoes.

In the present experiment, all fruit samples underwent identical COS pre-treatment, and parallel non-COS control groups under each light condition were not included. Therefore, our dataset cannot statistically separate the independent biological effects of COS versus light factors, nor can we quantify potential interactive or synergistic effects between COS and LED light signals. All comparisons in this manuscript reflect differences among varied light-quality/light-intensity treatments against a uniform COS-primed background. Future factorial experiments crossing ± COS factor with multiple light regimes will be required to disentangle main effects and interaction terms.

## 4. Materials and Methods

### 4.1. Experimental Materials and Screening Requirements

**Figure 10:**
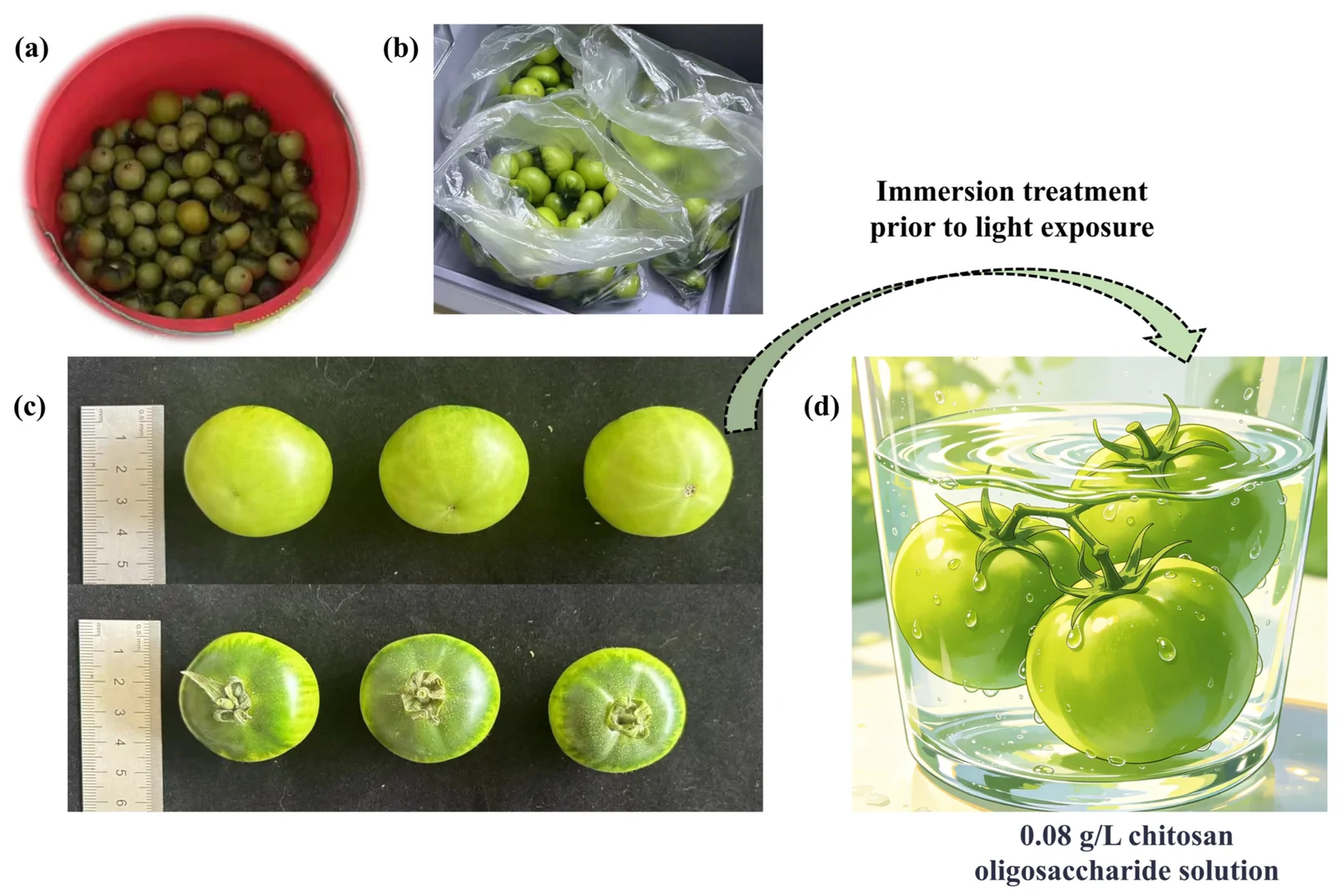
Azsaccharide immersion pre-treatment of green tomato materials. (**a**) Harvesting state of ‘Fen Junmei’ tomatoes at the mature-green stage. (**b**) After harvesting, transport the fruit immediately to the laboratory in a temperature- and humidity-controlled preservation container. (**c**) Initial phenotypic appearance of ‘Fen Junmei’ tomatoes at the mature-green stage prior to experimental treatment. (**d**) The selected tomatoes immersed in 0.08 g/L chitosan oligosaccharide solution for 1 h in a room-temperature environment. During the preparation of Figure 10d in this manuscript, the authors used "doubao", https://www.doubao.com/chat/create-image?channel=gdt_sem (accessed on 20 October 2026), version of Seedream 4.5 for the purposes of generation of Figure 10d. The instruction used to generate Figure 10d was: Please create an anime-style image depicting some green tomato fruits submerged in a clear, colorless liquid.

Tomato (*Solanum lycopersicum*) “Fen Junmei” is selected from the Shouguang demonstration base of Shanghai Qiande Seed Industry Co., Ltd. (Shanghai, China). It produces medium- to small-sized fruits, weighing approximately 40 ± 5 g per fruit, turning bright red when fully mature. Newly bred tomato seedlings were grown naturally with natural pollination, without any external substance treatment. For fruit experiments, flowers were marked on the day of flowering to determine the stages of fruit development and maturation [89]. At 8:00 a.m. on 21 January 2026, when the tomato fruits in the greenhouse reached the mature-green stage (MG—fully developed green fruit, approximately 35 days after flowering, with no further increase in fruit size) [90], fruits of similar size and maturity were harvested and quickly transported back to the laboratory within 2 h using preservation containers. For subsequent experiments, tomatoes of uniform size, without surface wounds or damage, and with intact stems were selected (Figure 10a–c). The chitosan oligosaccharide pre-treatment solution is selected from Beijing Telafeng Agricultural Science and Technology Co., Ltd. (Beijing, China)–Wasa brand, an organic water-soluble fertilizer containing chitosan oligosaccharide. According to the product instructions, for foliar spraying on surface application on fruits and vegetables, dilute 1000 times and apply every 10–15 days.

### 4.2. Experimental Site and Environment

**Figure 11:**
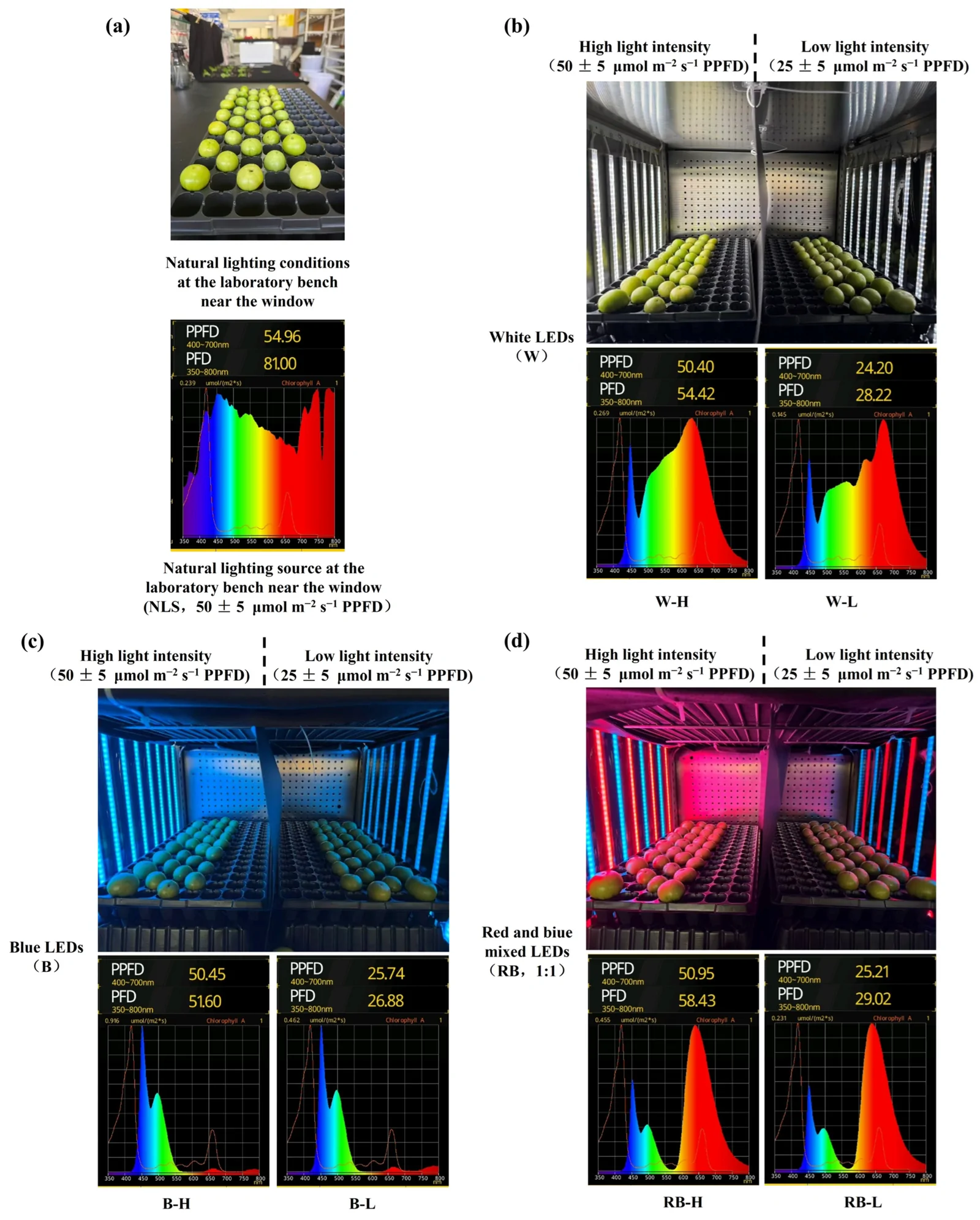
Experimental setup of multi-gradient light quality and light intensity regimes for postharvest elicitation of green tomatoes, with full-spectrum PPFD detection data for each light condition. (**a**) Ambient natural laboratory light source near the window (NLS); (**b**) white LED arrays set to high (W-H) and low (W-L) photosynthetic photon flux density (PPFD); (**c**) monochromatic blue LED light treatments at two intensity levels (B-H, B-L); (**d**) combined red–blue LED illumination (1:1 mixing ratio) with high (RB-H) and low (RB-L) light intensities. High/low light intensity: 50 ± 5/25 ± 5 μmol m^−2^ s^−1^ PPFD.

This experiment used the natural indoor light environment of northern China as a control. In the north (Shouguang, Weifang, China), during mid to late January, the indoor heating temperature during daytime reached 20–25 °C, while nighttime temperatures ranged from 15 to 20 °C, with a relative humidity of 60–65%. The average photosynthetic photon flux density (PPFD) of the natural light source (NLS) on the laboratory bench near the window, measured using the “five-point method,” was 50 ± 5 μmol m^−2^ s^−1^ (Figure 11a).

The experimental group was maintained in a plant growth chamber environment. The interior of the chamber was vertically divided into three levels, each containing an equal volume of plant cultivation chambers. On each level, two separate chambers were equally spaced on either side by 100% light-blocking black plastic shields. Thus, one chamber unit consisted of three levels with two chambers per level, totaling six chambers. These six chambers were completely isolated from one another to prevent light interference, yet allowed for air circulation, ensuring consistent environmental conditions such as temperature and humidity. On the right side of each cultivation chamber, a light-blocking film with 50% transmittance was used to reduce light intensity. The final light quality and intensity treatments were as follows (Figure 11b–d): from top to bottom, the first layer consisted of full-spectrum white light (white, W; left side high-intensity, W-H; right side low-intensity, W-L); monochromatic blue light (blue light, B; left side high-intensity, B-H; right side low-intensity, B-L); and red–blue mixed light (red/blue = 1:1, red and blue mixed lights, RB; left side high-intensity, RB-H; right side low-intensity RB-L). The high-light intensity was 50 ± 5 μmol m^−2^ s^−1^ PPFD, and the low-light intensity was 25 ± 5 μmol m^−2^ s^−1^ PPFD. The temperature inside the plant growth chamber was maintained between 15 and 20 °C, with a relative humidity of 60–65%.

### 4.3. Experimental Design

Before light treatment, mature-green tomatoes “Fen Junmei” were soaked in a 1000-fold diluted chitosan oligosaccharide organic water-soluble fertilizer solution (the effective component concentration of chitosan oligosaccharide is 0.08 g/L) for 1 h, then removed and air-dried at room temperature before proceeding with the subsequent 7-day light treatment experiment (Figure 10d). The control group was maintained under natural indoor lighting conditions throughout the 7-day experimental period. The experimental group, however, had tomatoes placed in plant growth chambers with different light qualities and intensities for 4 h each day from 17:00 to 21:00 (in northern China during winter, 17:00 corresponds to dusk when light intensity is relatively low), after which they were returned to the natural indoor environment. This procedure was repeated for seven days. To avoid variations in light exposure due to position differences, the positions of the tomatoes within each treatment group were randomly rearranged every morning at 8:00. All fruits received identical chitosan-oligosaccharide immersion pre-treatment prior to different light exposures, clearly establishing COS as the uniform baseline condition for all treatments; a “-COS” control group was not included in this experiment. Following processing, all tomato fruits—except those reserved for photographic documentation and fresh weight measurement—were manually deseeded, and the pericarp tissue was carefully excised to remove gel and placental tissues. Subsequently, uniform peel samples (comprising exocarp and adjacent mesocarp) were collected from the equatorial region of each fruit and immediately flash-frozen in liquid nitrogen before storage at −80 °C. The experiment employed a completely randomized design, with seven treatments each represented by 30 independent biological replicates (210 fruits total).

### 4.4. Measurement Method

The nine fresh tomatoes used in Section 4.4.1, Section 4.4.2, Section 4.4.3 and Section 4.4.4 are the same nine tomatoes.

#### 4.4.1. Observation of External Color and Recording of Days Required from Green Maturity to Color Break Stage

During the 7-day treatment period, nine fresh tomato fruits were marked for each treatment to monitor color changes. Three tomatoes with consistent color development were selected daily for visual assessment and photography. The stage when color transitioned from green to light yellow was considered the onset of the breaking-color phase. The average number of days required for tomatoes in each treatment to progress from the initial “mature-green” stage to the breaking-color phase was calculated.

#### 4.4.2. Measuring Fruit Surface Color

On day 7 of the experiment, the external color indices of tomato fruits under different treatments were measured using a DS-700E colorimeter (CaiPu Technology Co., Ltd., Hangzhou, China) (Table 1). Nine fresh fruits were randomly selected from each treatment, and the L value (lightness; higher values indicate greater brightness), a value (red saturation; higher values indicate deeper red), and b value (yellow saturation; higher values indicate deeper yellow) were determined at four equatorial positions on the fruit skin. A higher chroma value C indicates easier coloring. The hue angle H (°) represents a comprehensive color indicator, ranging from 0° to 180°, corresponding sequentially to magenta, red, orange, yellow, yellow–green, green, and blue–green: H = 0° for magenta, H = 90° for yellow, and H = 180° for blue–green. The calculations for C and H are shown in Equations (1) and (2), respectively [91].

(1) $$C/\%=\sqrt{a^{2}+b^{2}}$$

(2) H/(°) = tan^−1^ [(b/a) × (180/Π)]

#### 4.4.3. Determination of Tomato Fruit Firmness

After 7 days of treatment, nine fresh tomatoes were taken from each treatment group and their fruit firmness was evaluated using a texture analyzer (CT3, Brookfield Inc., Middleboro, MA, USA). In the puncture test, a cylindrical probe with a diameter of 2 mm was inserted 7 mm deep into the equatorial region of each fruit, and the maximum force value (peak) was recorded.

#### 4.4.4. Determination of Tomato Fruit Flavor Quality

After 7 days of treatment, nine fresh tomatoes were taken from each treatment group to measure flavor-related parameters using the Pocket Brix-Acidity Meter Master Kit PAL-BXIACID1 (ATAGO Co., Tokyo, Japan; measurement range: soluble solids 0.0–25.0%, titratable acidity 0.00–2.00 g/100 mL). Soluble solids content (refractive index value): at least 0.3 mL of juice was squeezed from the equatorial region and placed on the refractometer prism; after calibration with distilled water, the reading was recorded. Titratable acidity (expressed as citric acid equivalent): the juice was diluted 50-fold, and at least 0.3 mL was dispensed into the measuring port for analysis. Sugar–acid ratio: calculated as the ratio of soluble solids content to titratable acidity.

#### 4.4.5. Determination of Carotenoid and Anthocyanin Content

After 7 days of treatment, nine samples of tomato tissue stored at −80 °C were taken from each treatment group to analyze the carotenoid and anthocyanin content in tomato fruits. The procedure for carotenoid content analysis followed the instructions provided in the “Plant Carotenoid Content Detection Kit” manual (Catalog No.: BC4330, Solarbio, Beijing, China). The procedure for anthocyanin content analysis followed the instructions in the “Plant Anthocyanin Content Detection Kit” manual (Catalog No.: BC1380, Solarbio, Beijing, China).

### 4.5. Transcriptome Data Analysis

Samples used for transcriptome and metabolome analysis were collected from tomato fruits seven days after treatment. Given that the W-L and RB-H treatment groups showed the most significant differences in ripening progression, and due to budget constraints, this study prioritized four treatment groups—W-H, W-L, RB-H, and RB-L—for multi-omics analysis, aiming to systematically elucidate the mechanisms by which light quality and intensity regulate postharvest ripening in green-ripe tomatoes.

#### 4.5.1. RNA Extraction, cDNA Library Construction, and Sequencing

The total RNA was extracted from the samples of W-H, W-L, RB-H, and RB-L using a pBIOZOL Kit (BioFlux, Ahmedabad, India, BSC55ML) according to the protocol. The RNA templates used for our qRT-PCR validation were the same biological replicate fruit samples, separate from the samples used for RNA-seq transcriptome sequencing. The RNA concentration and RNA integrity number (RIN) were detected by a Qubit™ 4 Fluorometer (Thermo Scientific, Waltham, MA, USA) and a Bio-Fragment Analyzer (Qsep400, Bioptic, Taiwan, China), respectively. The RNA-seq libraries were constructed using the NEBNext Ultra RNA Library Prep Kit and then sequenced using the NovaSeq 6000 platform (Illumina, San Diego, CA, USA).

#### 4.5.2. Bioinformatics Analyses

Subsequently, the obtained raw reads were subjected for remove the primer/adaptor contaminations and paired reads featuring with more than 10% unknown nucleotides (Ns) and low base rating quality (Q-value ≤ 20) less than 50% by using FASTQ pre-processor [92]. The reference genome and its annotation files were downloaded from a specified website. HISAT was used to build an index, and clean reads were aligned to the reference genome [93,94]. StringTie was adopted as a computational method for assembling the mapped reads of each sample [95]. Novel genes were predicted and annotated by the DIAMOND (double index alignment of next-generation sequencing data) program [96].

Then, the expression levels of all transcripts were estimated by fragments per kilobase of transcript per million mapped fragments (FPKM), which could also be used to reflect the overall distribution of sample gene expression. The differential expression analysis between the two groups was conducted with DESeq2 [97]. The differentially expressed genes (DEGs) were selected on the basis of the criteria of |log_2_Fold Change| ≥ 1 and *p* value < 0.05. Afterwards, the Gene Ontology (GO) and Kyoto Encyclopedia of Genes and Genomes (KEGG) enrichment analyses of all the DEGs were performed using Metware Cloud tools (https://cloud.metware.cn) to ascertain their putative functions.

### 4.6. Metabolome Profiling Analysis

#### 4.6.1. Sample Preparation and Extraction

##### Solid Samples Class II

Materials were harvested, weighed, immediately frozen in liquid nitrogen, and stored at −80 °C until needed. Samples were ground to a powder in liquid nitrogen. An amount of 500 mg of the sample was transferred immediately to a 20 mL headspace vial (Agilent, Palo Alto, CA, USA), containing 2 mL NaCl-saturated solution to inhibit any enzyme reaction. The vials were sealed using crimp-top caps with TFE-silicone headspace septa (Agilent). At the time of SPME analysis, each vial was placed at 60 °C for 5 min, then an SPME Arrow (Agilent) of 120 µm DVB/CWR/PDMS was exposed to the headspace of the sample for 15 min at 60 °C.

##### GC-MS Conditions

After sampling, desorption of the VOCs from the SPME Arrow coating was carried out in the injection port of the GC apparatus (Model 8890; Agilent) at 250 °C for 5 min. The identification and quantification of VOCs were carried out using an Agilent Model 8890 GC and a 7000D mass spectrometer (Agilent), equipped with a 30 m × 0.25 mm × 0.25 μm DB-5MS (5% phenyl-polymethylsiloxane) capillary column. Helium was used as the carrier gas at a linear velocity of 1.2 mL/min. The injector temperature was kept at 250 °C. The oven temperature was programmed from 40 °C (3.5 min), increasing at 10 °C/min to 100 °C, at 7 °C/min to 180 °C, at 25 °C/min to 280 °C, and held for 5 min. Mass spectra were recorded in electron impact (EI) ionization mode at 70 eV. The quadrupole mass detector, ion source and transfer line temperatures were set, respectively, at 150, 230 and 280 °C. The MS was operated in selected ion monitoring (SIM) mode for the identification and quantification of analytes.

#### 4.6.2. Metabolite Data Analysis

##### PCA

Unsupervised PCA (principal component analysis) was performed using the statistics function prcomp within R version 4.5.3 (www.r-project.org) [98]. The data were processed by log\(_{2}\) transformation and zero-centered before PCA.

##### Hierarchical Cluster Analysis and Pearson Correlation Coefficients

The HCA (hierarchical cluster analysis) results of samples and metabolites were presented as heatmaps with dendrograms, while Pearson correlation coefficients (PCC) between samples were calculated by the cor function in R and presented only as heatmaps. Both HCA and PCC were carried out using the R package ComplexHeatmap. For HCA, normalized signal intensities of metabolites (unit variance scaling) are visualized as a color spectrum.

##### Differential Metabolites Selected

For two-group analysis, differential metabolites were determined by VIP (VIP > 1) and absolute Log_2_FC (|Log_2_FC| ≥ 1.0). For multi-group analysis, differential metabolites were determined by VIP (VIP > 1) and *p*-value (*p*-value < 0.05, ANOVA). VIP values were extracted from OPLS-DA result, which also contain score plots and permutation plots, was generated using R package MetaboAnalystR. The data were processed by log\(_{2}\) transformation and UV (unit variance scaling) before OPLS-DA. In order to avoid overfitting, a permutation test (200 permutations) was performed.

##### KEGG Annotation and Enrichment Analysis

Identified metabolites were annotated using the KEGG Compound database (http://www.kegg.jp/kegg/compound/ (accessed on 20 April 2026)), and annotated metabolites were then mapped to the KEGG Pathway database (http://www.kegg.jp/kegg/pathway.html (accessed on 20 April 2026)) [99].

### 4.7. Integrated Correlation Analysis of Transcriptome and Metabolome

The Pearson correlation coefficients were determined using the R package (cor function) according to the fold change in DEGs and DAMs. The correlation coefficients (value > 0.80 or <−0.80, and *p* < 0.05) were selected in the network plot for clarity.

### 4.8. qRT-PCR Validation of DEGs

For validation of the transcriptomic data from high-throughput sequencing, 16 DEGs involved in amino acid metabolism, terpenoid biosynthesis, flavonoid biosynthesis, fatty acid synthesis, ethylene synthesis, etc., were selected and quantified using the quantitative real-time PCR method (qRT-PCR). The gene-specific primers were all presented in Table S5. The procedures can be found in our previous publication [100]. Three biological replicates of each reaction were conducted and two *Actin* genes of tomato (*Solanum lycopersicum*) were selected as reference genes. After obtaining the Ct values, the relative transcript levels were finally calculated using the 2^−ΔΔct^ method [101].

### 4.9. Statistical Analysis and Graphs

The presented data of physiological parts, RNA-seq, metabolome experiment, and qRT-PCR were generated from no less than 3 biological replicates (*n* ≥ 3). The significant differences were determined by Duncan’s multiple range test (one-way ANOVA) at *p* = 0.05 with SAS v8.2 software (SAS Institute Inc., Cary, NC, USA). The bar graphs were plotted in GraphPad Prism (8.0.2 Ver, GraphPad procedure, Boston, MA, USA). The PCA (principal component analysis) graph was created by Metware Cloud tools (https://cloud.metware.cn).

## 5. Conclusions

This study characterizes how light quality and light intensity modify postharvest quality attributes in chitosan-oligosaccharide-pre-treated mature-green tomatoes, demonstrating that light quality exerts stronger regulatory priority than light intensity. Among the treatments, high-intensity red–blue composite light (50 μmol m^−2^ s^−1^ PPFD, RB-H) was found to be optimal. This treatment effectively shortened the ripening period of mature-green tomatoes, promoted the accumulation of carotenoids and anthocyanins, enhanced fruit skin coloration, reduced fruit firmness, and increased soluble solids content and sugar-to-acid ratio, thereby comprehensively improving both appearance and basic flavor quality. In contrast, low-intensity white light (W-L) significantly inhibited fruit ripening and nutrient accumulation, resulting in the poorest overall quality. Metabolomic analysis revealed that 870 volatile organic compounds were detected in tomato fruits, with terpenes being the core aromatic components. RB-H treatment significantly upregulated the accumulation of over a hundred volatile compounds associated with fruit, floral, and sweet aromas, reshaping the sensory profile of tomato aroma and substantially enhancing flavor complexity. Transcriptome screening identified six major light-responsive core transcription factor families: WRKY, MYB, AP2/ERF, bHLH, NAC, and C2H2. WGCNA revealed two key co-expression modules—green and magenta—closely associated with tomato ripening and aroma metabolism, which respond specifically to light quality and light intensity, respectively. Within these modules, 16 core hub genes showed specific upregulation under RB-H treatment and play crucial roles in transmitting light signals to downstream metabolic pathways. KEGG pathway enrichment analysis confirmed that terpenoid backbone biosynthesis, monoterpenoid biosynthesis, sesquiterpenoid and triterpenoid biosynthesis, and tyrosine metabolism are the core pathways involved in light-regulated characteristic aroma formation in tomatoes. RB-H treatment upregulates the expression of key rate-limiting enzyme-encoding genes in these pathways, providing sufficient precursors for the synthesis of various volatile compounds, thereby elucidating at the transcriptional level the molecular mechanism by which red–blue high-intensity light improves tomato flavor quality. In summary, this study evaluates postharvest quality improvement via high-intensity red–blue LED supplemental lighting applied to chitosan-oligosaccharide-pre-treated tomatoes, and revealed the complete regulatory network involving “light signal–transcription factor–co-expression module–terpenoid/tyrosine metabolism–aromatic compound accumulation.” This provides important theoretical foundations and practical production solutions for precise LED light environment control and targeted improvement of fruit flavor and quality in greenhouse tomatoes.

## Figures and Tables

**Table 1:** Color parameters of tomatoes under different treatments.

Treatment	*L* (Lightness)	*a* (Red–Green Axis)	*B* (Yellow–Blue Axis)	*C* (Chroma)	*H°* (Hue Angle)
NLS	48.93 ± 2.85 ab	18.70 ± 1.36 b	11.10 ± 1.22 bc	37.68 ± 1.42 b	35.67 ± 2.06 c
W-H	51.78 ± 3.41 a	17.34 ± 1.72 bc	14.36 ± 0.95 ab	35.33 ± 2.94 bc	51.77 ± 0.57 ab
W-L	48.35 ± 3.43 ab	16.17 ± 1.17 c	15.31 ± 0.97 a	32.57 ± 2.57 c	52.32 ± 0.55 a
B-H	51.69 ± 2.59 a	19.01 ± 1.67 b	11.02 ± 1.31 bc	38.27 ± 1.36 b	47.46 ± 1.49 b
B-L	51.12 ± 2.10 a	18.75 ± 1.41 b	12.32 ± 1.13 b	38.16 ± 1.24 b	47.88 ± 1.56 b
RB-H	49.11 ± 2.56 ab	22.55 ± 1.27 a	6.28 ± 1.54 d	43.41 ± 1.70 a	21.35 ± 1.97 d
RB-L	49.02 ± 2.76 ab	18.61 ± 1.27 b	9.29 ± 1.27 c	41.78 ± 1.63 ab	34.39 ± 2.35 c

Lightness (*L*), red-green Axis (*a*), yellow-blue Axis (*B*), chroma (*C*) and hue angle (*H*°) values of tomatoes subjected to different processing treatments. *L* value (brightness): higher values indicate greater brightness; *a* value (redness): higher values indicate deeper red tones; *B* value (yellowness): higher values indicate deeper yellow tones; Chroma (*C*): the higher the value, the easier it is to color; Hue angle (*H*°): a comprehensive color indicator ranging from 0° to 180°, sequentially representing magenta, red, orange, yellow, yellow-green, green, and blue-green; i.e., *H *= 0° corresponds to magenta; *H* = 90° corresponds to yellow; *H* = 180° corresponds to blue-green. Different lowercase letters indicate significant separation among treatments by Duncan’s multiple range test at *p* ≤ 0.05. Vertical bars represent means ± standard error (*n* = 9).

## Data Availability

The original contributions presented in this study are included in the article. Further inquiries can be directed to the corresponding authors.

## Supplementary Materials

The following supporting information can be downloaded at: https://www.mdpi.com/article/10.3390/ijms27219555/s1.
